# Pseudovirus-Based Neutralization Assays as Customizable and Scalable Tools for Serological Surveillance and Immune Profiling

**DOI:** 10.3390/pathogens14111129

**Published:** 2025-11-06

**Authors:** Caio Bidueira Denani, Bruno Pimenta Setatino, Denise Pereira, Ingrid Siciliano Horbach, Adriana Souza Azevedo, Gabriela Coutinho, Clara Lucy Ferroco, Janaína Xavier, Robson Leite, Ewerton Santos, Maria de Lourdes Maia, Waleska Dias Schwarcz, Ivanildo Pedro Sousa

**Affiliations:** 1 Laboratório de Análise Imunomolecular, Instituto de Tecnologia em Imunobiológicos (Bio-Manguinhos), Fundação Oswaldo Cruz, Rio de Janeiro 21040-360, Brazil; caio.denani@bio.fiocruz.br (C.B.D.); bruno.setatino@bio.fiocruz.br (B.P.S.); denisesg.pereira@fiocruz.br (D.P.); ingrid.horbach@bio.fiocruz.br (I.S.H.); adriana.soares@bio.fiocruz.br (A.S.A.); 2 Programa de Pós-Graduação em Medicina Tropical, Instituto Oswaldo Cruz (IOC), Fundação Oswaldo Cruz, Rio de Janeiro 21040-360, Brazil; 3 Departamento de Desenvolvimento Experimental e Pré-Clínico (DEDEP), Instituto de Tecnologia em Imunobiológicos (Bio-Manguinhos), Fundação Oswaldo Cruz, Rio de Janeiro 21040-360, Brazil; gabriela.coutinho@bio.fiocruz.br; 4 Departamento de Assuntos Médicos, Estudos Clínicos e Vigilância Pós-Registro (DEAME), Instituto de Tecnologia em Imunobiológicos (Bio-Manguinhos), Fundação Oswaldo Cruz, Rio de Janeiro 21040-360, Brazil; clara.vasconcellos@bio.fiocruz.br (C.L.F.); janaina.xavier@bio.fiocruz.br (J.X.); robson.cruz@bio.fiocruz.br (R.L.); ewerton.santos@bio.fiocruz.br (E.S.); lourdes.maia@bio.fiocruz.br (M.d.L.M.); 5 Laboratório de Virologia e Parasitologia Molecular, Instituto Oswaldo Cruz (IOC), Fundação Oswaldo Cruz, Rio de Janeiro 21040-360, Brazil

**Keywords:** pseudovirus, SARS-CoV-2, neutralizing antibody (nAb), PRNT, serological surveillance

## Abstract

Neutralizing antibodies (nAbs) are key indicators of protection against SARS-CoV-2, and their measurement remains essential for monitoring vaccine responses and population immunity. While the plaque reduction neutralization test (PRNT) is the gold standard, it relies on replicative viruses and is not suited for high-throughput applications. Here, both an in-house and a commercial pseudovirus-based neutralization (PBN) assay were standardized and compared with PRNT to assess performance and concordance. The in-house PBN employed a VSV-ΔG pseudovirus encoding NanoLuc and displaying the SARS-CoV-2 Spike from the Wuhan or Omicron BA.1 variants in HEK293T-hACE2 cells, whereas the commercial assay (Integral Molecular, Philadelphia, PA, USA) used a lentiviral backbone with Renilla or GFP reporters and Wuhan or Omicron XBB.1.5/XBB.1.9 Spikes in Vero E6-ACE2-TMPRSS2 cells. Both assays showed strong correlations with PRNT, the commercial assay; moreover, they offered superior reproducibility and scalability, while the in-house version provided a cost-effective alternative suitable for BSL-2 settings. A total of 600 serum samples from vaccinated individuals were analyzed by commercial PBN at collection time points, from pre-vaccination to twelve months post–second dose, enabling large-scale screening, revealing marked differences in neutralization between Wuhan and Omicron XBB.1.5/1.9, and allowing unbiased classification of low, medium, and high responders using k-means clustering. The geometric mean titers (log_10_ GMT) highlighted a ~1.5 log_10_ (eightfold) reduction in neutralizing activity against Omicron, reflecting antibody waning and antigenic drift. Altogether, this study integrates assay standardization, PRNT comparison, and large-scale immune profiling, establishing a robust framework for harmonized pseudovirus-based neutralization testing.

## 1. Introduction

Coronavirus disease 2019 (COVID-19), caused by the severe acute respiratory syndrome coronavirus 2 (SARS-CoV-2), spread rapidly worldwide and, according to the WHO COVID-19 Epidemiological Update, resulted in more than seven million deaths [1]. Beyond the immense human and economic impact, the pandemic highlighted the critical role of seroneutralization tests. Alongside PCR for diagnosis, the Plaque Reduction Neutralization Test (PRNT) is considered the reference standard and has been crucial in monitoring neutralizing antibodies (nAbs) and assessing immune protection after natural infection [2,3] or vaccination [2,3,4].

While some debate remains regarding the correlation between PRNT-measured nAbs and protective immunity [5], multiple studies on different viruses [6,7,8,9,10,11], including SARS-CoV-2 [12] consistently demonstrate that nAbs are reliable correlates of protection, given their ability to block viral entry. These antibodies can persist for months or years and are associated with lower reinfection rates [13,14,15,16] and milder disease, particularly in severe COVID-19 cases [17]. In contrast, binding antibody assays, though valuable for initial screening, may overestimate protection as they do not necessarily reflect functional neutralization [17].

Despite its reliability, PRNT remains limited for large-scale or multicenter studies due to its laborious, time-consuming, and low-throughput nature [18,19]. It also applies only to plaque-forming viruses, restricting its use for non-cytopathic or inconsistently plaque-forming agents [20,21]. Furthermore, differences in cell lines and culture conditions can alter plaque morphology, reducing reproducibility [22,23]. In addition, for some viruses—such as SARS-CoV-2 during the pandemics—PRNT involves biosafety level 3 (BSL-3) containment, which further limits its throughput and turnaround time.

A major limitation of PRNT is the manual counting of plaques—a slow, error-prone procedure requiring significant personnel time. Although WHO has historically recommended manual quantification [18], this approach is subjective and susceptible to human error, potentially compromising data integrity and adherence to Good Laboratory (GLP) and Clinical Laboratory Practices (GCLP).

To overcome these limitations, alternative assays have been developed over the years, such as the micro-neutralization test (MNT) [5,6,7], microPRNT [8,9], focus reduction neutralization test (FRNT) [10,11,12], virus reduction neutralization test (VRNT) [13], immune-plaque assay-FRNT (iPA-FRNT) [14], and fluorescence reduction neutralization test (FluoRNT) [15]. However, all these methods require the use of infectious viruses, demanding containment in BSL-3 or BSL-4 laboratories, depending on the pathogen. This restricts access, increases operational costs, and limits throughput in the context of emerging viral threats like SARS-CoV-2.

Among the most promising alternatives, tests that do not require infectious particles offer several advantages, such as the surrogate virus neutralization test (sVNT) [16,17] and the reporter virus neutralization assay (RVNA), also known as PBN assay [24,25,26].

PBN assays employ replication-incompetent viral particles pseudotyped with envelope proteins of interest and have emerged as a safe and efficient alternative to conventional methods. When working with infectious viruses that require BSL-3 containment, PBN assays can be conducted under BSL-2 conditions, thereby reducing biosafety constraints while enhancing reproducibility and scalability [23]. Furthermore, their modular design allows for rapid adaptation; for instance, replacing the plasmid encoding the Wuhan SARS-CoV-2 Spike with a variant-specific Spike (e.g., XBB) enables the evaluation of neutralizing responses against emerging strains [24].

**Figure 1:**
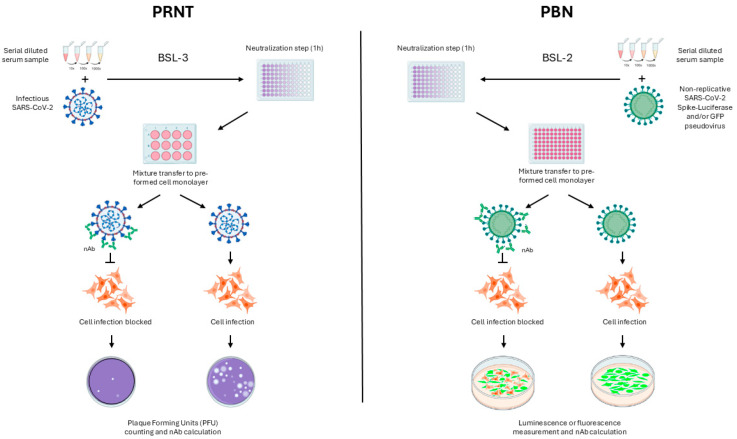
Schematic comparison of PRNT and PBN assay principles. PRNT quantifies neutralizing antibodies by measuring the reduction in viral plaques formed, also referred to as Plaque Forming Unit (PFU), after infection with infectious SARS-CoV-2. In contrast, PBN detects reporter gene expression (luciferase or GFP) in cells infected by non-replicative pseudoviruses, allowing quantification through luminescent or fluorescent signals. Compared to PRNT, which typically requires 3–4 days for plaque visualization under BSL-3 conditions and is performed in low throughput formats (e.g., 6–24 well-plates), PBN assays offer faster readouts (24–72 h), can be conducted under BSL-2 conditions, and are compatible with high throughput formats such as 96 well-plates, enabling greater testing capacity.

Regarding these tests’ principles, PRNT quantifies nAbs by measuring the reduction in viral plaques formed in cell monolayers exposed to replicative viruses. In contrast, pseudovirus-based neutralization (PBN) assays detect reporter gene expressions (e.g., luciferase or GFP) in cells infected by replication-incompetent pseudoviruses, allowing quantification of neutralization through luminescent or fluorescent signals (Figure 1).

The flexibility of pseudotyping also enables the development of models for other viruses—using NS1 or envelope proteins for Dengue [27], HA or NA for Influenza [28,29], or GP for Ebola [30]. Despite being used for decades [31], the COVID-19 pandemic refocused attention to PBN assays, with many groups adapting or developing protocols to correlate their results with PRNT or MNT [32,33,34].

Despite broad application, few studies have achieved robust standardization of PBN methods, including the use of well-characterized reference sera, definition of a plug-and-play assay architecture, clinical sample cohort testing, and demonstration of equivalence with PRNT under rigorous conditions. Standardization of serological assays is crucial to ensure the reliability and safety of test results, reducing the likelihood of errors such as false positives or false negatives. Additionally, it supports reproducibility, which is vital for both clinical and non-clinical settings, including vaccine development and epidemiological surveillance. In this context, the present study sought to standardize two complementary PBN systems—one commercial and one developed in-house—to assess their comparability to PRNT and evaluate consistency, reproducibility, scalability, and cost-effectiveness across assay backbones and cell models for routine immunomonitoring, aiming to deliver a reliable, customizable, and scalable platform that can complement the gold standard test in vaccination studies and public health surveillance.

## 2. Materials and Methods

### 2.1. Cell Lineages

HEK293T cells (ATCC, Manassas, VA, USA—#CRL-3216™) were acquired from Fisher Scientific, Waltham, MA, USA—#50-238-3588) and used to produce the in-house Vesicular Stomatitis Virus (VSV) pseudotyped particles. These cells are a derivative of human embryonic kidney 293 (HEK293) cells, stably transfected with the SV40 large T antigen to enhance plasmid replication and protein expression [35].

HEK293T-hsACE2 cells (Integral Molecular, Philadelphia, PA, USA—#C-HA102) were used during the seroneutralization assays with the commercial pseudotyped viruses. HEK293T-hsACE2 cells, derived from the human embryonic kidney 293T (HEK293T) cell line, and similarly to Vero E6-ACE2-TMPRSS2, were stably transduced with a lentiviral vector encoding the human ACE2 receptor under the control of the CMV promoter. Following transduction, cells were selected using puromycin to establish stable expression of hACE2, enabling efficient SARS-CoV-2 Spike-mediated viral entry [36].

Vero E6-ACE2-TMPRSS2 cells were kindly provided by Prof. Amilcar Tanuri (Federal University of Rio de Janeiro) for the seroneutralization assays with the in-house pseudotyped viruses. This lineage was obtained from the National Institute of Biological Standards and Control (NIBSC, South Mimms, Hertfordshire, UK—#101003) and derived from the parental African green monkey kidney epithelial cell line Vero E6 (ATCC #CRL-1586™) and was stably transduced with a bicistronic expression construct encoding human ACE2 and TMPRSS2 under the control of the CAG promoter. The cells were selected using puromycin to ensure stable co-expression of both SARS-CoV-2 entry factors [34].

### 2.2. Pseudotyped Viruses

To safely and consistently assess neutralizing activity, two pseudovirus-based systems were used: a commercial lentiviral platform (Integral Molecular, USA) and an in-house system based on vesicular stomatitis virus (VSV). Both detection systems employ synthetic, non-replicative viral particles engineered to express the SARS-CoV-2 Spike protein on their envelope, enabling measurement of viral entry inhibition without handling infectious SARS-CoV-2. The construction of these synthetic viruses involves molecular cloning of the Spike gene into plasmids encoding structural proteins of the respective viral backbone (lentivirus or VSV), followed by co-transfection in permissive producer cells (HEK293T). The resulting pseudoviruses carry a reporter gene (Luciferase or GFP), which allows quantification of neutralization through luminescence or fluorescence readouts.

Commercial Wuhan and Omicron XBB 1.5/1.1.9 pseudotyped viruses, carrying Luciferase (#RVP-702L and #RVP-786L, Integral Molecular—USA) or GFP (#RVP-786G, Integral Molecular—USA) reporter genes were adopted to standardize the commercial-based PBN assay. The particles are based on a Lentiviral (LV) core, Wuhan or Omicron XBB Spike protein on the surface, and Renilla-Luciferase or GFP reporter gene in the genome. Particles containing the GFP reporter gene were used to monitor the pseudotyped virus entry into the host cells and to define the best time to be adopted as hours post-infection (hpi), aiming for the highest reporter signal. For this purpose, DAPI (1 µg/mL—Invitrogen, Carlsbad, CA, USA) was used for nuclear staining, and images were acquired using an EVOS M5000 fluorescence microscope (ThermoFisher Scientific, Waltham, MA, USA). As a rigorously defined negative control for viral entry, GFP pseudoviruses were pre-incubated for 1 h at 37 °C with a serum sample from previously characterized by PRNT and the standardized commercial PBN assay as strongly neutralizing. The serum sample, referred as High nAb Titer (HnAbT), was chosen among the samples from the clinical study “Analysis of the Immune Response to Drug, Biopharmaceutical, and Vaccine Testing through Functional In Vitro Studies Using Human Primary Cells”, described in Titer-Stratified Panel of Sera samples session. The resulting pseudovirus–serum mixture was then applied to HEK293T-hACE2 cells under the same experimental conditions as the testing pseudovirus. The absence of GFP fluorescence in this condition confirmed the inhibition of viral entry and validated the assay’s ability to discriminate between productive infection and neutralization-mediated blockage.

In-house–developed pseudotyped viruses, kindly provided by Dr. Luciana Jesus da Costa (Federal University of Rio de Janeiro), were engineered with a vesicular stomatitis virus (VSV) core, the SARS-CoV-2 Spike protein (Wuhan or Omicron BA.1) on the surface, and the NanoLuc luciferase reporter gene. Their generation followed a two-step process using a two-plasmid system: one plasmid encoded the VSV-G core proteins fused to the NanoLuc reporter (provided by the CRO company Nexelis, Laval, QC, Canada), and the other contained the full-length SARS-CoV-2 Spike sequence from either the ancestral Wuhan strain or the Omicron BA.1 variant (provided by Dr. Paul Bieniasz from Rockfeller University). Plasmid design, transfection conditions, and post-production handling were based on optimized protocols described by Whitt (2010), Havranek et al. (2020) and Schmidt et al., 2020 [37,38,39]. In the first stage, HEK293T-hACE2 cells were transfected with the VSV-G/NanoLuc plasmid to produce VSV particles expressing the G glycoprotein and carrying the NanoLuc genome. These particles were then used in the second stage, to infect newly transfected HEK293T-hACE2 cells expressing the SARS-CoV-2 Spike (Wuhan or BA.1), leading to the generation of pseudoviruses bearing Spike on their envelope and lacking the G gene. Supernatants containing pseudoviruses were harvested, clarified through 0.2 µm PVDF filters and stored at −80 °C until use. The resulting replication-deficient ΔG-VSV particles displayed the SARS-CoV-2 Spike on their surface and the NanoLuc reporter in their genome, ensuring single-cycle infectivity. Both Wuhan and BA.1 pseudoviruses produced robust luminescent signals and were efficiently neutralized by reference sera, confirming proper Spike incorporation and functional suitability for neutralization assays.

### 2.3. Titer-Stratified Panel of Sera Samples

In the scope of the clinical study “Analysis of the Immune Response to Drug, Biopharmaceutical, and Vaccine Testing through Functional In Vitro Studies Using Human Primary Cells”, the FIOCRUZ Ethics Committee approved the procedures related to sample collection (Certificates of Ethical Appreciation Submission—CAAE #34728920.4.0000.5262, #30846920.7.0000.0008, and #15120613.4.0000.5262) and all participants authorized the use of their samples, and the methods employed were carried out following the relevant norms and guidelines.

After the approval, sera samples from the participants were collected, inactivated in 56 °C water bath for 30 min, and screened by in-house validated Wuhan or Omicron BA.1 SARS-CoV-2 PRNT [4]. Samples’ nAb titers were categorized in 4 groups statistically defined, according to PRNT cut-off established by our group during the assay validation [4]: negative (≤14), positive low (100 ≥ titer > 14), positive medium (500 ≥ titer > 100), and positive high (titer > 500). The sera panel, consisting of 40 samples (10 for each group), was used to compare PBN assays to each other and against the gold standard PRNT.

### 2.4. Control Sera Definition

Serum samples from the same study (Analysis of the Immune Response to Drug, Biopharmaceutical, and Vaccine Testing through Functional In Vitro Studies Using Human Primary Cells) had their nAb titer evaluated to define sera control to be used as quality control for each PBN assay. Samples were screened by commercial and in-house PBN assays to identify nAb titers that fit into the categories defined by a titer-stratified panel of SARS-CoV-2 PRNT. As a result, negative, positive low, positive medium, and positive high titer samples were defined as control sera for the Wuhan and Omicron XBB commercial assay and the Wuhan and Omicron BA.1 in-house assay. The definitions of the strains were based on the virus circulation at the time of sample collection.

### 2.5. PBN Protocol Definition

Neutralization assays employed HEK 293T-hsACE2 or Vero-hsACE2 cells and serum samples as analytes. Serum samples were serially diluted (1:10 to 1:31,250) in DMEM high glucose (#D6546, Sigma-Aldrich-Merck, St. Louis, MO, USA) supplemented with 5% FBS, 1% gentamicin, and 0.4% amphotericin B (#SBR00045, Sigma-Aldrich—Merk). In all assays, equal volumes of pseudovirus (10^4^ RLU for Wuhan LV; 10^5^ RLU for XBB LV; 10^5^ RLU for Wuhan VSV; 10^6^ RLU for BA.1 VSV) and inactivated sera were added to each well before being incubated for 1 h at 37 °C for the neutralization step. The mixture was then transferred to 96-well plates containing pre-seeded target cells. Plates were incubated for 72 h in LV-based assays and 24 h in VSV assays.

The activity of luciferase was measured using GloMax luminometer (Promega, Madison, WI, USA). For commercial particles, cells were lysed before reading; for in-house VSV pseudoviruses, the NanoLuc signal was detected directly from the supernatant using One-Glo reagent (Promega—USA). Neutralization titers (PBN_50_) were determined by interpolating the serum dilution resulting in 50% inhibition of pseudovirus entry compared to viral control (VC). Titers > 1:10 were considered positive.

### 2.6. Comparison Between PBN Assays and PRNT

The PBN assays using commercial or in-house developed pseudoviruses had their equivalence assessed. For this purpose, each assay using Wuhan pseudotyped viruses was used to quantify nAb titer values from the titer-stratified panel of sera samples. The distribution normality of the titer values generated by both PBN assays for each sample was assessed by the Shapiro–Wilk test, and the titer values were compared by the Wilcoxon paired test. Statistical analyses were performed in GraphPad Prism software version 10.3.1.

Similar procedure was adopted to compare PBN assays with PRNT: titers generated by commercial (Wuhan) and in-house (Wuhan and Omicron BA.1) assays were compared to PRNT titers for the same SARS-CoV-2 strains. The Shapiro–Wilk test was used to determine the normal distribution of titer values, which were then compared using the Wilcoxon paired test. Statistical analyses were performed in GraphPad Prism software version 10.3.1.

### 2.7. Screening of Clinical Cohort

Samples from the clinical study “Immunogenicity Study of the COVID-19 Vaccine (Recombinant)—Fiocruz/AstraZeneca—Administered with a Four- or Eight-Week Interval Between Doses (VacCov4-8s),” conducted by the Department of Medical Affairs, Clinical Studies, and Post-Marketing Surveillance (DEAME) (Bio-Manguinhos—Fiocruz) were evaluated using the commercial PBN assay. The study has been approved by the Brazilian National Research Ethics Commission (CONEP) (substantiated opinion nº 7.242.045). A total of 600 blood serum samples from 100 study participants were collected at 6 timepoints, according to the study protocol. Collection points 1 to 6 represent the groups pre-vaccination (1), 28 days after the 1st dose (2), 28 days after the 2nd dose (3), Pre-3rd dose (4), 28 days after the 3rd dose (5), and 12 months after the 2nd dose (6). Besides screening the 600 blood serum samples by PBN assay, a subset of 240 of these samples was statistically designed to be tested by PRNT: 40 samples were randomly selected among the total of 100 for each collection point. Testing this subset by both assays was planned to reinforce the assessment of PBN-PRNT equivalence.

### 2.8. Defining Numeric Ranges for the nAb Titer Categories

It was desirable that the samples identified as positive by the standardized PBN assay were further categorized according to their neutralizing antibody titer values into specific ranges. The number of these categories was determined in a data-driven manner to optimize clustering while minimizing biases associated with arbitrary threshold selection.

For this purpose, internal cluster validity indices were evaluated across a range of candidate k values (k = 2–6). Specifically, the average Silhouette width (where higher values indicate better-defined clusters) and the Davies–Bouldin index (where lower values indicate greater cluster separation) were computed for each k. The final number of clusters was selected based on the configuration that simultaneously maximized the Silhouette coefficient and minimized the Davies–Bouldin index. These indices are well-established and widely used for assessing clustering quality [40,41], and their combined use has been recommended in comparative studies to enhance robustness in determining the optimal number of clusters [42].

The Silhouette index assesses how well each observation is assigned to its respective cluster by comparing the intra-cluster cohesion with the separation from the nearest neighboring cluster. The average silhouette is the mean of the silhouette coefficient s(i) across all observations. Higher values indicate a more well-defined and robust clustering structure: Values close to +1 suggest that the observation is well-clustered and clearly separated from other clusters. Values near 0 indicate that the observation lies on the boundary between clusters. Negative values imply potential misclassification, suggesting the observation might belong to a different cluster.

The Davies–Bouldin index evaluates clustering quality by comparing the internal compactness of each cluster with its external separation from other clusters. Lower values indicate better clustering performance. In this study, raw data from the titles were used without transformations to preserve the natural distribution and variation patterns inherent to the original data scale. This approach maintains mathematical relationships and facilitates direct interpretation of the resulting clusters, ensuring fidelity to the actual patterns observed in the sample.

After defining the best category number (k), samples classification was performed using the k-means clustering algorithm [43], an unsupervised machine learning method that partitions data into k groups (in this case, k = 3) according to the similarity among observations. This approach optimizes cluster formation by minimizing intra-cluster variance and maximizing inter-cluster separation. Additionally, this method enables a data-driven categorization of samples, avoiding the need for arbitrary cutoff values. Clustering was applied to the original dataset, which included 600 values from the Omicron test and 600 from the Wuhan test. This strategy ensures greater consistency in sample classification and facilitates subsequent comparative analyses.

### 2.9. Statistical Analysis

All statistical analyses were performed using GraphPad Prism software (version 10.0; GraphPad Software, San Diego, CA, USA). Neutralizing antibody titers were log_10_-transformed prior to analysis. Correlations between assays (in-house PBN, commercial PBN, and PRNT) were evaluated using Spearman’s rank correlation coefficient (ρ) with 95% confidence intervals (CI95%). Comparisons between methods (e.g., PRNTPRNT vs. commercial PBN; PRNT vs. in-house PBN) were conducted using the Mann–Whitney U test for two independent groups. For samples collected at multiple time points, the Friedman test was applied, and when significant differences were detected, pairwise comparisons were performed using the Nemenyi post hoc test to compare the first collection with each subsequent time point. Geometric mean titers (GMTs) and their corresponding 95% confidence intervals were calculated for each group. Categorical distributions of samples within neutralization titer ranges were expressed as percentages. The significance level was set at *p* < 0.05 for all comparisons. Finally, cluster analysis (K-means) was performed in R software (version 4.3.2; R Foundation for Statistical Computing, Viena, Austria) using the “stats” package, to stratify samples into low, intermediate, and high neutralization groups based on log_10_-transformed titers. Prior to clustering, the optimal number of categories (k) was determined by evaluating internal validation indices, including the Silhouette coefficient and the Davies–Bouldin index, to identify the configuration that best balanced cluster cohesion and separation, minimizing arbitrary threshold selection.

**Figure 2:**
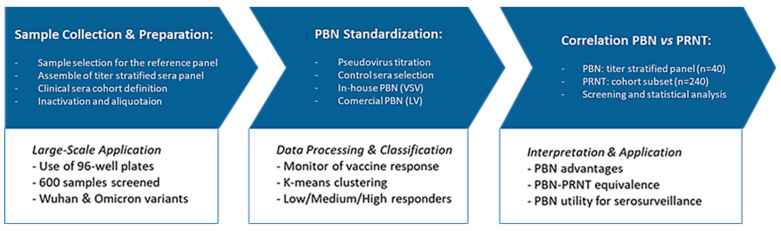
Workflow of the methodological steps of the study. The process was divided into three main phases: (1) Sample collection and preparation, (2) Standardization of the pseudovirus-based neutralization assay (PBN), and (3) Correlation between PBN and PRNT assays.

The material, equipment, and methods used and standardized during this work are categorized below, and an equivalent workflow that summarizes them is exhibited in Figure 2.

## 3. Results

### 3.1. Standardization of Pseudovirus-Based Neutralization (PBN) Assays

#### 3.1.1. Entry of GFP-XBB-SARS-CoV-2 Pseudovirus into HEK293T-ACE2 Cells

**Figure 3:**
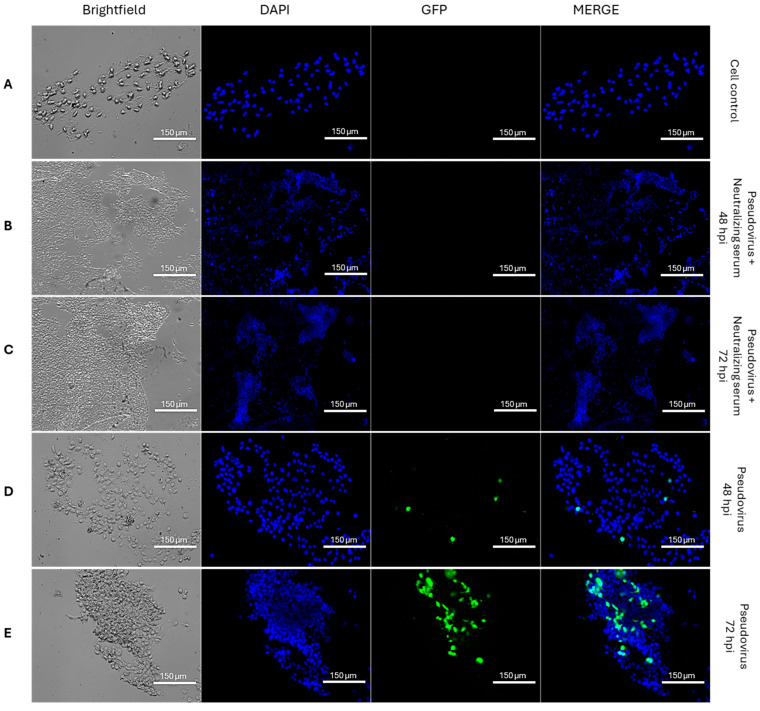
Micrographs showing brightfield (BF), nuclear staining (DAPI), and GFP signals from HEK293T-hACE2 cells exposed to GFP-SARS-CoV-2-XBB.1.5 pseudoviruses. Cells were either mock-treated (**A**) or infected and imaged at 48 hpi and 72 hpi after 1 h preincubation with a known neutralizing serum (HnAbT; **B**,**C**) or with medium alone (**D**,**E**). Fluorescence was acquired using an EVOS M5000 microscope (ThermoFisher Scientific, USA) after DAPI staining (1 µg/mL; Invitrogen, USA). Representative images from three independent experiments are shown at 10× (**B**,**C**) and 20× magnification (**A**,**D**,**E**).

To assess pseudovirus entry and transgene expression over time, fluorescence microscopy was performed on HEK293T-hACE2 cells infected with lentiviral particles pseudotyped with the SARS-CoV-2 XBB.1.5 Spike and encoding GFP as a reporter gene. Images were acquired in brightfield, DAPI, and GFP channels, and subsequently merged to evaluate colocalization. In mock-infected cells (Figure 3A), only DAPI fluorescence was observed, with no detectable GFP signal.

To validate assay specificity and provide a negative control for viral entry, GFP-pseudovirus was pre-incubated with a serum sample previously characterized as strongly neutralizing by PRNT and by standardized commercial PBN (HnAbT). Following incubation, the pseudovirus–serum mixture was applied to HEK293T-hACE2 cells. In this condition, no GFP fluorescence was detected at either 48 (Figure 3B) or 72 h post-infection (hpi) (Figure 3C), confirming that neutralization effectively prevented pseudovirus entry and transgene expression.

In contrast, cells infected with untreated pseudovirus exhibited increasing GFP fluorescence over time. At 48 hpi, isolated GFP-positive cells were visible, indicating successful viral entry and initiation of reporter expression (Figure 3D). By 72 hpi, a marked increase in both the number and intensity of fluorescent cells was observed (Figure 3E), demonstrating progressive pseudovirus uptake and transcriptional activity. These results confirm that 72 hpi is the optimal time point for quantifying pseudovirus-mediated transduction in this cellular model.

#### 3.1.2. Selection of Reference Sera for Quality Control of the Luc-SARS-CoV-2 Pseudovirus Assays

**Figure 4:**
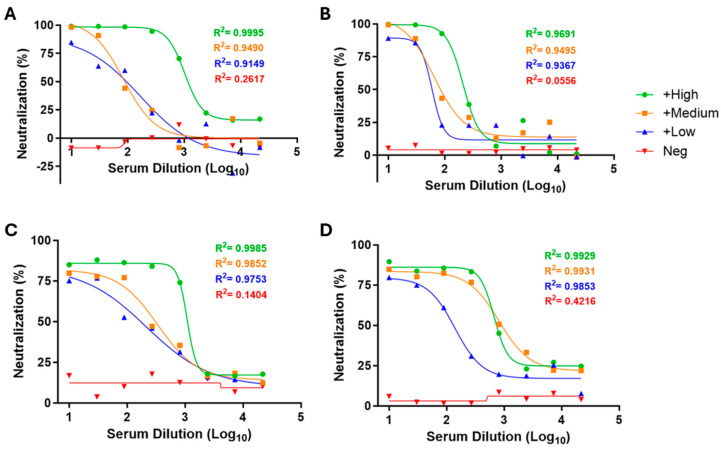
Standardization of reference sera for PBN assays using commercial and in-house developed pseudoviruses. Graphs (**A**,**B**) (commercial PBN) and (**C**,**D**) (in-house PBN) represent the neutralization profiles of sera candidates during their selection, respectively, for Wuhan (**A**,**C**) and Omicron XBB (**B**,**D**). Neutralization profiles are represented for negative (red curve), positive low (blue curve), positive medium (orange curve), and positive high (green curve) sera. The nonlinear regression was plotted and R^2^ calculated by a sigmoidal dose–response fit curve with variable slope in GraphPad Prism version 10.3.1.

Neutralization assays usually adopt reference sera to establish quality control over time. Accordingly, sera from the in-house validated SARS-CoV-2 PRNT [4] were evaluated by commercial (Figure 4A,B) and in-house (Figure 4C,D) pseudovirus-based neutralization test as control candidates. The tested samples—specific to each pseudovirus strain—displayed neutralization profiles consistent with predefined categories: negative serum (no detectable or very low neutralizing activity), and positive sera exhibiting low, intermediate, or high levels of neutralizing antibody titers (Figure 4). This stratification allowed the selection of representative control samples across a dynamic range of neutralizing capacities. The consistency and reproducibility of these profiles across replicate experiments support their use as reference standards to monitor assay performance and ensure analytical reliability.

#### 3.1.3. Comparison Between Commercial and In-House PBN Tests

**Figure 5:**
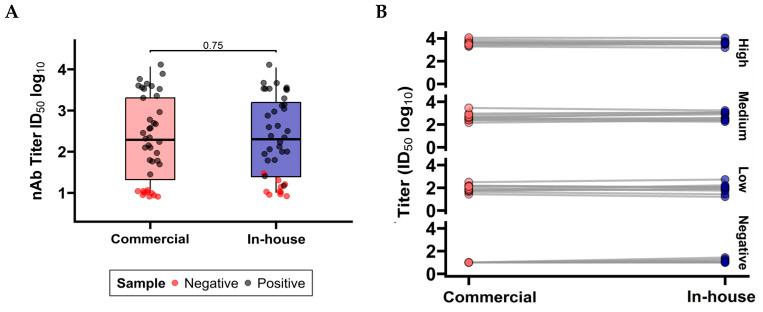
Comparison between PBN methods (commercial and in-house), testing by both assays, 40 samples screened previously by Wuhan-PRNT and categorized into samples with negative titer, positive high titer, positive medium titer, and positive low titer. Graph (**A**) depicts that samples classified as positive by PRNT are plotted in black and those classified as negative are red, with the Wilcoxon test for independent samples (Mann–Whitney) used to confirm the difference between the two distributions. Graph (**B**) shows the differences between values generated by each test. *p* = 0.75 (α = 0.05); therefore, the null hypothesis that the distributions are equal is not rejected. Statistical analysis was performed using GraphPad Prism 10.3.1.

In addition to being compared to the gold-standard PRNT, the commercial and in-house pseudovirus-based neutralization (PBN) assays were evaluated for their ability to measure neutralizing antibody (nAb) titers. This evaluation used the stratified serum panel previously characterized by PRNT against the SARS-CoV-2 Wuhan strain [4]. The Mann–Whitney-Wilcoxon test for independent samples was used to assess the distributions of nAb titers obtained by each assay (Figure 5A), and Figure 5B depicts the differences in titer values generated by each test for each sample. In both analyses, the null hypothesis—stating that the median difference between methods is zero—could not be rejected, supporting the equivalence of the commercial and in-house PBN assays in measuring neutralizing antibody responses.

#### 3.1.4. Screening of PRNT Titter-Stratified Panel by Standardized SARS-CoV-2 PBN Assays

Pseudovirus-based neutralization assays were performed using both in-house and commercial pseudoviral particles expressing the SARS-CoV-2 Spike protein to evaluate the neutralizing activity of serum samples previously characterized by PRNT. The inclusion of sera with known neutralization titers, previously quantified using a statistically validated PRNT protocol [4], enabled a direct comparison between the pseudovirus-based assays and the gold-standard method. This approach aimed to assess the concordance and performance of the alternative pseudovirus assays in discriminating samples across a range of neutralizing capacities, thereby supporting their potential application in routine serological monitoring.

Regarding the commercial pseudovirus system LV-Luc-SARS-CoV-2, the evaluation was limited to the serum panel previously tested against the ancestral SARS-CoV-2 Wuhan strain, due to the absence of a matched panel validated by PRNT for the Omicron XBB variant. In contrast, the in-house pseudovirus system allowed for broader comparative analysis, including serum panels previously assessed by PRNT using both the Wuhan strain and the Omicron BA.1 variant. This experimental design allowed us to evaluate the flexibility and relevance of in-house pseudovirus assays in recapitulating PRNT-based neutralization data across distinct SARS-CoV-2 variants.

**Figure 6:**
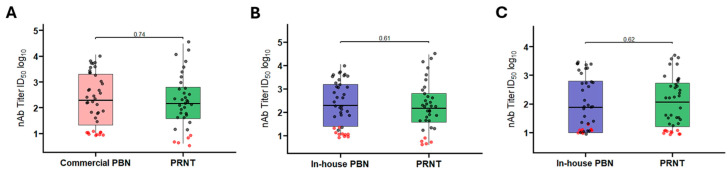
Figures illustrate nAb titer regions occupied by the samples categorized by PRNT screening and tested by Luc-SARS-CoV-2 PBN assays. Graph (**A**) shows titer distributions from both commercial PBN (light pink) and PRNT (green) for Wuhan (*p*-value = 0.74), while graphs (**B**,**C**) refer, respectively, to Wuhan (*p*-value = 0.61) and BA.1 (*p*-value = 0.62) in-house PBN assays (purple) compared to their corresponding PRNT measurements (green). In (**A**–**C**), samples classified as positive by PRNT are plotted in black and those classified as negative are red. The Mann–Whitney test was adopted to compare titer distributions, and no statistically significant difference was found (>alpha = 0.05); therefore, the null hypothesis that the distributions are equal is not rejected. Statistical analysis performed in GraphPad Prism version 10.3.1.

The distribution of positive neutralizing antibody titters in both the commercial (Figure 6A) and in-house PBN assays (Figure 6B,C), was similar to those obtained by the PRNT for the corresponding serum samples. This lack of statistically significant differences supports the robustness and reliability of the PBN assays in reproducing PRNT-derived classifications across distinct viral variants and platforms.

### 3.2. Evaluation of Neutralizing Antibody Responses in a Clinical Cohort Using the Commercial PBN Assays

A cohort of 600 serum samples from the VacCov4-8s study was screened using the standardized commercial PBN to evaluate the presence and levels of nAbs across vaccination dose intervals, as well as the immune protection status induced by the SARS-CoV-2 Fiocruz/AstraZeneca vaccine.

**Figure 7:**
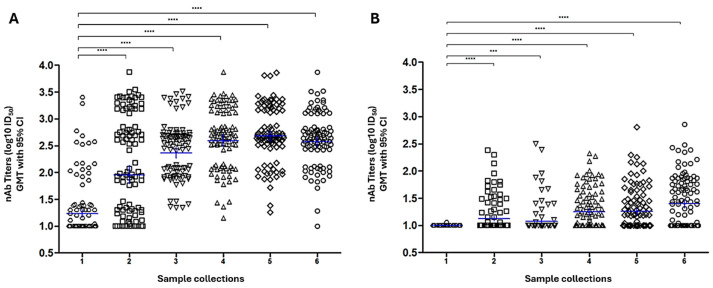
Application of commercial PBN to screen a serum cohort. Graphs (**A**,**B**) illustrate the nAb titer distributions and the GMT (blue bars) of the 100-sample serum cohort sera along 6 collection timepoints from a vaccine clinical study for Wuhan and XBB 1.5, respectively. Collection points 1 to 6 represent the groups Pre-vaccination (1), 28 days after the first dose (2), 28 days after the second dose (3), Pre third dose (4), 28 days after the third dose (5), and 12 months after the second dose (6). Pre-test: Shapiro–Wilk normality test was performed, and it was not significant (alpha = 0.05). Test Friedman tested equality among time collections, and it was rejected: (*p* < 0.001) ***; Post-hoc test: pairwise Wilcoxon tested difference between each collection time pair (*p* < 0.05); *** *p* < 0.001; **** *p* < 0.0001. GraphPad Prism 5 was used to produce the graph and perform the statistical analysis.

The screening revealed that GMT against the Wuhan started with 1.23 log_10_ ID_50_ in collection one, increased to about 2–2.36 log_10_ ID_50_ in collections two and three, then plateaued at values around 2.65 log_10_ ID_50_ across collections four, five, and six (Figure 7A). In contrast, modest titers were detected against the Omicron XBB strain across the six collection points. Although there was a significant difference between the points—highlighted by the asterisks—GMT increased somewhat (1.21 to 1.38 log_10_ ID_50_) between collections four and six (Figure 7B).

The cohort screening revealed nAb titer profiles in participant samples, confirming the vaccine’s potential to elicit a humoral response primarily against the Wuhan strain, as well as the test’s ability to detect a wide range of titer values. This confirms the assay potential for immunological surveillance during vaccine development and clinical trials. Notably, the serum panel used for this evaluation was collected before Omicron XBB circulation. This temporal mismatch may partially account for the lower neutralization titers observed against this variant, reflecting limited immunological exposure at the time of sample collection.

Finally, to confirm the concordance between the gold standard PRNT and the standardized commercial PBN assays, a subset of 240 serum samples—originally screened by PBN assays using Wuhan and Omicron XBB.1.5 pseudoviruses—was selected for further testing by PRNT against the corresponding SARS-CoV-2 strains. This subset enabled direct comparison of neutralizing antibody titers obtained by both methodologies for each viral variant.

**Figure 8:**
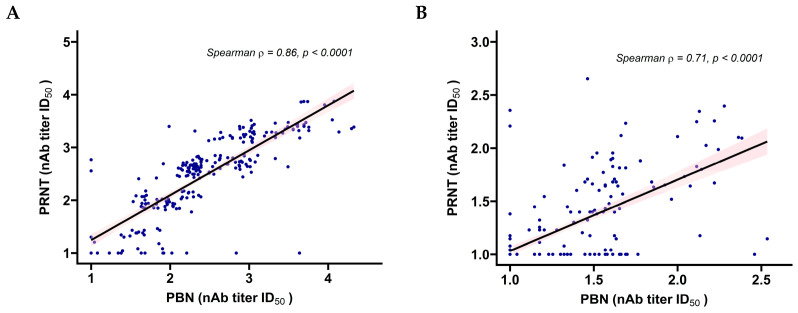
Correlation between neutralizing antibody titers obtained by standardized PBN assays and gold standard PRNT for a subset of 240 clinical serum samples. (**A**): Wuhan strain. (**B**): Omicron XBB.1.5 variant. Titers were Log10-transformed and analyzed using Spearman’s rank correlation coefficient (ρ). A moderate-to-strong and highly significant monotonic correlation was observed for Omicron XBB.1.5 (ρ = 0.71, *p* < 0.001), while a very strong and highly significant correlation was observed for Wuhan (ρ = 0.86, *p* < 0.001). GraphPad Prism 5 was used to produce the graph and perform the statistical analysis.

Neutralizing antibody titers measured by PBN and PRNT in Wuhan and Omicron XBB.1.5, were transformed to a Log10 scale and correlated using Spearman’s rank correlation coefficient (ρ). This non-parametric test evaluates the strength and direction of monotonic associations, regardless of linear relationships. The resulting correlations revealed a consistent and positive association between the two assays, both for Wuhan (Figure 8A) and Omicron XBB 1.5 (Figure 8B), confirming the reliability of the standardized commercial PBN test to accurately quantify neutralizing antibodies in clinical samples across distinct SARS-CoV-2 variants.

Negative samples were defined as those with neutralizing antibody titers equal to or lower than 10 (≤1 log_10_), as serum samples were first diluted at 1:10. Positive samples showed the following log10 titer ranges: Low: 1.04 to 2.87 (corresponding to titers between 11 and 741); Medium: 2.87 to 3.54 (titers between 742 and 3484); and High: more than 3.54 (titers >3484).

**Figure 9:**
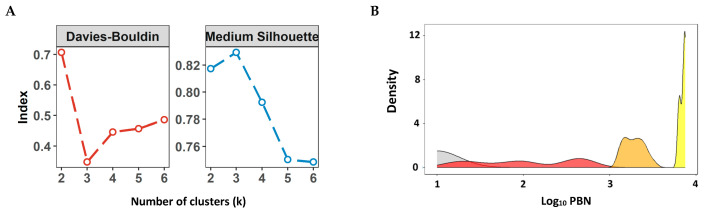

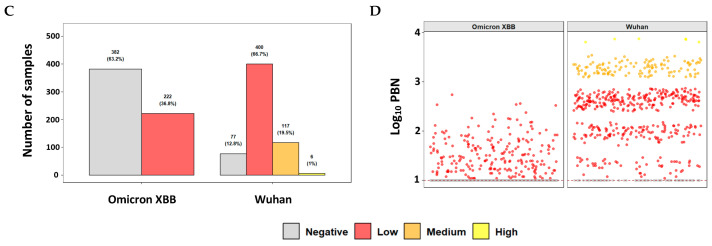
(**A**) Left and right panels show, respectively, the Davies–Bouldin and Silhouette indices used to identify the optimal number of clusters (k = 3). (**B**) Density plot of log_10_-transformed PBN titers from all 1200 positive samples (600 Wuhan and 600 Omicron XBB.1.5). The multimodal distribution supports stratification into three titer categories—low (red), medium (orange), and high (yellow)—as determined by k-means clustering—with color segmentation highlighting the clustered category range. The gray-shaded area represents negative samples (log_10_ PBN ≤ 1). (**C**) A Bar plot showing the distribution of samples across four categories (negative, low, medium, and high) by variant. Most Omicron XBB.1.5 samples were classified as negative (63.7%) or low (33.1%), with a few medium- (3.6%) and no high-titer samples. In contrast, Wuhan samples were more evenly distributed, with 12.8% negative, 21.3% low, 45.3% medium, and 20.5% high. (**D**) Dot plot of log_10_ PBN titers for each sample, grouped by variant and colored according to classification. Clustering reveals distinct serological profiles between Wuhan and Omicron XBB.1.5, highlighting the reduced neutralization against XBB.1.5.

To determine the optimal number of clusters, both the Silhouette and Davies–Bouldin indices were applied for values of k (number of clusters) ranging from 2 to 6. The results indicated that k = 3 provides the best balance between the two indices, with a Silhouette score of 0.829 (indicating strong cluster structure) and a Davies–Bouldin index of 0.348 (indicating well-separated and compact clusters). Graphs from Figure 9A indicate the optimum number of cluster (k) calculated by Davies-Bouldin (left) and Silhouette (right) indices. The density plot (Figure 9B) illustrates the total distribution of nAb titers obtained by PBN using data from both Wuhan and Omicron XBB samples together. Color segmentation highlights the category ranges determined by clustering. The multimodal shape of the curves, suggesting the data separation into three groups and confirmed by Silhouette and Davies-Bouldin indices, aligns with the natural structure of the dataset, indicating the suitability of the clustering approach. Additionally, the minimal overlap of density curves, especially between medium and high categories, implies a consistent separation among the titer profiles.

Figure 9C displays the categorical distribution of samples by variant. For Omicron XBB, all samples were classified as negative (63%) or low (37%), and none defined as medium and high. Conversely, For the Wuhan variant, although a small proportion of samples (1%) exhibited high positive nAb titers, the majority (~67%) showed low positive titers. Additionally, a subset of samples was classified as having medium positive titers (~19%), while a smaller fraction (~13%) was categorized as negative. The dot plot (Figure 9D) depicts the distribution of individual samples across the groups (negative, low, medium, and high), separated by variant. For Omicron XBB, samples were exclusively classified as negative or positive low, reflecting a weaker immune response against this variant. In contrast, the Wuhan strain exhibited a higher proportion of samples in the positive low and medium categories, indicating generally stronger nAb titers. Negative samples (≤1 log_10_) were detected in both variants, whereas Omicron XBB had a higher frequence, reinforcing the pattern of reduced serological response against this lineage at the moment of sample collection.

## 4. Discussion

In this study, we standardized and validated PBN assays—both in-house and commercial—for quantifying SARS-CoV-2 nAbs and compared them to the gold standard PRNT. Furthermore, we applied commercial PBN on a large cohort of clinical serum samples, aiming to assess its capacity to detect and discriminate nAb titers across a wide dynamic range.

We found high correlations between the in-house and commercial PBN assays, as well as between them and PRNT. Importantly, Spearman’s coefficients indicated that PBN tests may rank samples similarly to PRNT, even across different variants. Nevertheless, despite the significant association, absolute nAb titer values varied between assays, which was predicted given that the measurements were obtained from two different biological approaches and consistent with previous studies using different neutralization platforms [2,44].

Intrinsic differences in assay principles may be the cause of the greater divergence between PRNT and PBN titers observed in higher ranges. PRNT quantifies neutralization by directly preventing plaque formation of replication-competent virus. This often requires higher antibody concentration to fully inhibit multiple rounds of infection. In contrast, PBN assays are single-cycle systems that measure inhibition of viral entry. As a result, neutralization curves may plateau earlier and, at high antibody concentrations, titers may be lower. Moreover, this mismatch may be amplified at higher neutralization levels due to differences in pseudovirus backbones, Spike protein density on the viral surface, and assay readouts (cytopathic effect vs. reporter signal). Together, these points highlight the continued need for harmonized reference standards and diagnostic infrastructure to ensure comparability across studies and consistency in vaccine evaluation

Based on commercial PBN data for both Wuhan and XBB strains, we applied k-means clustering to categorize samples into low, intermediate, and high nAb levels in order to objectively stratify nAb responses. To determine the optimal number of clusters, we employed two widely used clustering validation metrics: the Silhouette and Davies–Bouldin indices. The analysis indicated that k = 3 provided the best balance between intra-cluster cohesion and inter-cluster separation, with a Silhouette score of 0.829 and a Davies–Bouldin index of 0.348, supporting the presence of a well-defined and compact cluster structure. This approach minimizes within-group variability and maximizes between-group separation, avoiding arbitrary cutoffs and ensures internal consistency.

The resulting stratification revealed expected immunological patterns, such as higher titers in vaccinated individuals and lower responses in participants tested more than one-year post-vaccination. Additionally, it also enabled the identification of atypical cases with elevated baseline titers, possibly due to an unrecognized prior infection. The density plot (Figure 9B) supports the clustering outcome, showing a multimodal distribution of nAb titers with minimal overlap between categories, especially between medium and high groups. This reinforces the suitability of the clustering approach. Furthermore, the categorical distribution by variant (Figure 9C) highlights the reduced serological response against Omicron XBB, with most samples classified as negative or low, in contrast to the broader distribution observed for the Wuhan strain. This analytical framework complements the methodological standardization described above, reinforcing the robustness of our findings despite the acknowledged limitations.

Our findings also reveal that commercial PBN assay effectively distinguished samples across these categories and presented low nonspecific background at low dilutions. This aligns with studies showing that assay specificity may be harmed by background reactivity in negative sera, especially when Spike protein is only target [45,46]. Interestingly, the commercial PBN assay appeared to be more specific for the Wuhan pseudovirus, as indicated in Figure 7 and Figure 9. Since strong antibody responses typically result in steeper neutralization curves and higher endpoint titers in pseudovirus systems, it is plausible that samples with higher neutralizing antibody titers against the ancestral strain may highlight differences in assay performance. This effect may reflect the higher affinity of vaccine-induced antibodies for epitopes preserved in the Wuhan Spike protein and the methodological sensitivity of the lentiviral platform in detecting inhibition at high antibody concentrations. Conversely, the reduced neutralization observed for Omicron XBB may be partially explained by the fact that this variant was not yet circulating when the serum samples were collected, limiting the presence of variant-specific antibodies in the cohort. This underscores a common challenge in variant-specific neutralization studies, where the timing of sample collection can impact the representativeness of immune responses. These results underline the need for careful interpretation when comparing across strains and imply that, although the commercial PBN is robust for large-scale applications, its performance may be affected by the magnitude of antibody responses and the antigenic distance between variants.

Most previously published studies with pseudovirus-based neutralization assays have been limited either to small standardization panels or to the evaluation of a single viral strain, often the ancestral Wuhan variant [33,47]. In contrast, our work expands on these findings by incorporating both large-scale clinical screening (*n* = 600) and cross-variant assessment including Omicron XBB.1.5/XBB.1.9, thereby providing a more comprehensive evaluation of assay performance across antigenically distant strains. Another distinctive feature of our study is the application of k-means clustering for nAb stratification. While most reports rely on predefined cutoffs or arbitrary thresholds [47,48], our approach offers a data-driven method that ensures internal consistency and improves comparability across studies. This not only addresses the lack of standardization in defining protective titters but also enables the detection of atypical responders that might otherwise be overlooked. Additionally, we have thoroughly investigated the differences found at higher antibody titers, while previous studies have highlighted only general correlations between PBN and PRNT [32,49]. Our findings suggest that such differences may arise from intrinsic assay properties and the biological characteristics of antibody–antigen interactions [50], adding nuance to the interpretation of equivalence between these platforms. Together, these contributions fill important gaps in the current literature by combining methodological standardization, large-scale clinical application, and refined data analysis strategies.

PBN assays provide several advantages including scalability, lower labor demands than PRNT, and compatibility with high-throughput analysis [33]. They can quickly adapt to emerging strains due to their ability to incorporate variant-specific Spike proteins. Our findings support their utility in measuring nAb responses to both ancestral (Wuhan) and immune-evasive variants such as Omicron XBB.1.5. Notably, the correlation between PBN and PRNT titers was slightly lower for XBB, possibly due to structural and epitope-level changes that impact antibody binding [46].

While high nAb titers have been associated with protection against symptomatic and severe COVID-19 [3,51], defining a universal threshold remains elusive due to differences in vaccine platforms, virus strains, and clinical outcomes [52]. Nonetheless, PBN assays represent a practical tool for large-scale surveillance and immunological profiling, with strong potential for complementary use alongside PRNT, particularly in times of high demand, when quick decisions are essential.

We also emphasize the importance of rigorous pseudovirus design. It is well established that differences in the density of Spike proteins between pseudoviruses and infectious virions may artificially inflate nAb titers [44]. In our work, pseudovirus production followed a carefully optimized plasmid ratio to ensure appropriate Spike incorporation, minimizing this interfering variable and improving assay reliability.

Finally, it is widely known that distinct serological assays serve complementary roles. While ELISA and sVNT are useful for preliminary screening and rapid monitoring [53,54], neutralization assays remain critical for functional assessment of protective immunity. Among seroneutralization tests, PBN has been demonstrating over the decades its importance—for example, in measuring neutralizing antibodies against Influenza A(H7N9) in outbreak settings under BSL-2 conditions [55] or in evaluating CHIKV immunity using pseudotyped CHIKV in human sera (Korean isolate) [56]. This foundation was reinforced during COVID-19 by its benefits and highlighted the key steps to be optimized during standardization to achieve equivalence with the gold standard.

Despite the strengths of our study, some limitations should be acknowledged. First, although we demonstrated strong correlations between PBN and PRNT, absolute neutralization titters vary between platforms, highlighting the persistent challenge of assay harmonization and the need for international reference standards. Second, while our cohort was large and diverse, it was restricted to samples derived from a single vaccine platform, which may limit the application of our findings to other immunization strategies or naturally infected populations. Third, we were unable to generate an in-house serum panel for testing with the commercial PBN against the XBB.1.5 pseudovirus, despite including both ancestral and Omicron variants. This would have strengthened the comparative analysis between platforms. Finally, as with all pseudovirus systems, neutralization readouts may be impacted by intrinsic differences in the viral backbone, Spike density, or entry mechanisms when compared to live viruses, underscoring the importance of continuous methodological improvement. Recognizing these caveats, our study nevertheless advances the field by introducing strategies that address some of these gaps.

In conclusion, our study supports the implementation of standardized PBN assays as robust alternatives to PRNT for the quantification of SARS-CoV-2 nAbs, with potential to be customized to other target viruses. Their versatility, reproducibility, and suitability for variant-specific surveillance make them valuable tools for immunological monitoring in clinical trials and public health programs, thereby reinforcing their role in advancing vaccine evaluation and immune surveillance strategies.

## Data Availability

The original data presented in this study will be made publicly available online in accordance with the journal’s policy. All datasets supporting the findings of this article will be accessible upon publication.
