# Correction: Denani et al. Pseudovirus-Based Neutralization Assays as Customizable and Scalable Tools for Serological Surveillance and Immune Profiling. *Pathogens* 2025, *14*, 1129

**DOI:** 10.3390/pathogens15020173

**Published:** 2026-02-05

**Authors:** Caio Bidueira Denani, Bruno Pimenta Setatino, Denise Pereira, Ingrid Siciliano Horbach, Adriana Souza Azevedo, Gabriela Coutinho, Clara Lucy Ferroco, Janaína Xavier, Robson Leite, Ewerton Santos, Maria de Lourdes Maia, Waleska Dias Schwarcz, Ivanildo Pedro Sousa

**Affiliations:** 1 Laboratório de Análise Imunomolecular, Instituto de Tecnologia em Imunobiológicos (Bio-Manguinhos), Fundação Oswaldo Cruz, Rio de Janeiro 21040-360, Brazil; caio.denani@bio.fiocruz.br (C.B.D.); bruno.setatino@bio.fiocruz.br (B.P.S.); denisesg.pereira@fiocruz.br (D.P.); ingrid.horbach@bio.fiocruz.br (I.S.H.); adriana.soares@bio.fiocruz.br (A.S.A.); 2 Programa de Pós-Graduação em Medicina Tropical, Instituto Oswaldo Cruz (IOC), Fundação Oswaldo Cruz, Rio de Janeiro 21040-360, Brazil; 3 Departamento de Desenvolvimento Experimental e Pré-Clínico (DEDEP), Instituto de Tecnologia em Imunobiológicos (Bio-Manguinhos), Fundação Oswaldo Cruz, Rio de Janeiro 21040-360, Brazil; gabriela.coutinho@bio.fiocruz.br; 4 Departamento de Assuntos Médicos, Estudos Clínicos e Vigilância Pós-Registro (DEAME), Instituto de Tecnologia em Imunobiológicos (Bio-Manguinhos), Fundação Oswaldo Cruz, Rio de Janeiro 21040-360, Brazil; clara.vasconcellos@bio.fiocruz.br (C.L.F.); janaina.xavier@bio.fiocruz.br (J.X.); robson.cruz@bio.fiocruz.br (R.L.); ewerton.santos@bio.fiocruz.br (E.S.); lourdes.maia@bio.fiocruz.br (M.d.L.M.); 5 Laboratório de Virologia e Parasitologia Molecular, Instituto Oswaldo Cruz (IOC), Fundação Oswaldo Cruz, Rio de Janeiro 21040-360, Brazil

There was an error in the original publication [1]. The mistake occurred during our final round of revisions in response to the reviewers’ comments. While reorganizing and updating the reference list, two citations were inadvertently omitted; these now correspond to the newly added References 18 and 19. This omission caused a shift in the numbering of several in-text citations and led to additional inconsistencies: Reference 35 was mistakenly assigned to the wrong article and has now been replaced with the correct source, and Reference 43 was incorrectly written and has been corrected. The insertion of these missing references, together with the correction of other reference entries, required the renumbering of several subsequent citations throughout the main text to maintain proper numerical order.

A correction has been made to the References section. The newly added references appear below:

18. World Health Organization. Guidelines for Plaque Reduction Neutralization Testing of Human Antibodies to Dengue Viruses. 2007. Available online: https://iris.who.int/handle/10665/69687 (accessed on 6 August 2025).
19. Denani, C.; Horbach, I.; Setatino, B.; Azevedo, A.; Lima, S.; Schwarcz, W.; Sousa, I. Evolution of the PRNT: Merging tradition and innovation to set the gold standard in the era of automation. *Hum. Vaccin. Immunother.* **2025**, *21*, 2528368. https://doi.org/10.1080/21645515.2025.2528368.

With this correction, the order of some references has been adjusted accordingly.

The authors would also like to update the Acknowledgments section. Unfortunately, they inadvertently omitted two important individuals. The corrected statement is as follows:

The authors would like to thank the technical staff of LANIM (Laboratório de Análises Imunológicas) for their valuable support during sample processing and experiment execution. We also express our appreciation to Beatriz Borges and Elena Caride for their important contributions to project management, particularly in resource coordination, oversight of project activities, and participation in result discussions, and to Bio-Manguinhos/Fiocruz for providing logistical support. We thank Luciana Jesus da Costa for the development of the in-house pseudoviruses and Amílcar Tanuri for kindly providing the HEK293T-hsACE2 cells used in this study. We also extend our sincere gratitude to Raissa Andrade for her medical-scientific leadership role in the clinical study, and to the Directors, Principal Investigators, and research teams from the clinical centers that enabled the collection and processing of samples for this study: Maria Vitória Hadland Seidl and Clarice Monteiro Vianna (UECI), Celia Menezes Cruz Marques (SMSRJ), Jorge Augusto Callado Afonso, Jaime Luís Lopes Rocha, Marcelo de Paula Loureiro, Meila Bastos de Almeida, Letícia Schiavo, and Rossana Baggio Simeoni (TECPAR), Pedro Ribeiro Barbosa (IBMP), Stenio Perdigão Fragoso (ICC/Fiocruz Paraná), Marion Burger (SMS Curitiba), and Carlos Eduardo Virgini Magalhães and Douglas Poshinger Figueiredo (Hospital Universitário Pedro Ernesto/UERJ). Their contributions were essential to the successful execution of the cohort study.

The authors state that the scientific conclusions are unaffected. This correction was approved by the Academic Editor. The original publication has also been updated.
